# Partner choice increases observed reciprocity-based cooperation but decreases unobserved stake-based cooperation

**DOI:** 10.1098/rsos.251654

**Published:** 2025-11-26

**Authors:** Pat Barclay

**Affiliations:** ^1^ Department of Psychology, University of Guelph, Guelph, Ontario, Canada

**Keywords:** reputation, partner switching, interdependence, stake, anonymity, relational mobility, observation, pseudoreciprocity, altruism, game theory

## Abstract

According to current theory and experiments, cooperation is more likely to evolve when organisms can choose to replace uncooperative partners with cooperative ones. However, there is a downside to this partner choice: when partners can be easily replaced, organisms have less stake in their partners’ welfare and will therefore be less likely to help keep those partners alive and well enough to reciprocate. Here, I present a mathematical model showing that when a third party is present, organisms will provide more observable help to their partners (reciprocity/signalling-based helping), but less anonymous help that would keep that partner in good condition (stake-based helping). The net effect of partner choice depends on the relative strength of these two factors: partner choice has a more positive effect if interactions are short (i.e. less stake), when observers judge based on observed helping (i.e. reputation matters), and when one can have multiple cooperative partners at the same time. These results show the importance of differentiating between helping that relies on observation (e.g. reciprocity and signalling), helping that requires no observation (e.g. kinship and stake), and how the two types interact.

## 1. Introduction

In the behavioural sciences, the traditional view is that partner choice is good for cooperation. When organisms can choose whom to interact with, cooperators can abandon defectors and assort instead with each other. This assortment selects for cooperation on two time scales: (i) on evolutionary time scales, as organisms with cooperative sentiment leave more offspring than organisms who defect and get ostracized (e.g. [1–13]); and (ii) within an organism’s lifespan, as defectors are forced to cooperate lest they be abandoned or ostracized (e.g. [14–21]). Partner choice can even cause an escalation of cooperation, because the most cooperative partners are preferentially chosen for social relationships, which creates an incentive to compete to be more cooperative than others (‘competitive helping’ [3,22–28]). Consequently, in societies where people can easily change partners (i.e. high ‘relational mobility’), people are more trusting, socially supportive, confident and happy [29,30].

However, there are less-studied downsides to partner choice. Recently, Stallen *et al*. [31] show that partner choice can create inequality, because more productive partners assort with each other to share their high productivity, and less productive partners are stuck with each other sharing low productivity. Another downside is that if organisms switch partners more often, this reduces the expected length of interactions, which previous research shows is crucial for reciprocity (e.g. [32,33]). Here, I focus on a similar but distinct third downside: when organisms can switch partners easily, they have less stake in the wellbeing of their existing partners.

### 1.1. Stake in a partner’s welfare

What does it mean to have a stake in a partner? When organism A does something that benefits organism B—intentionally or not—then B benefits from A’s continued presence and wellbeing. B would benefit from investing in A’s welfare so that A can continue to do whatever they do to benefit B (i.e. B has a stake in A’s welfare). This principle has been described many times under many names, including pseudoreciprocity [34], byproduct reciprocity [35], stake [36], partnership [37], group augmentation [38], irreplaceability [39], interdependence [40,41] and vested interests [42]. This principle even applies across species, or when one organism is unaware that it benefits the other. For example, a tree grows leaves for the self-interested reason of photosynthesizing, but if those leaves provide beneficial shade for the organisms underneath, then those shaded organisms have a vested interest in promoting the tree’s survival and growth so it can continue to provide that shade, even if the tree never ‘knows’ it was helped [43]. This is a key feature of stake-based helping: it does not require that the recipient observe the help and respond (i.e. it is not reciprocity); instead, the helper receives direct benefits from the recipient’s continued wellbeing.

Organisms can come to have a stake in another’s welfare in many ways. Monogamously mated organisms have a stake in their romantic partner for their future reproduction and parenting towards joint offspring, and affines (in-laws) have a stake in each other if they both care for kin that they share [44]. Group members may benefit from each other’s presence if larger groups provide more effective foraging, detection of predators or defence against other groups [38]. More generally, some organisms provide byproduct benefits for others as a function of their self-interested actions, like the aforementioned tree providing shade or oxygen. In any of these relationships, if one partner were threatened with injury, death or incapacitation, it would be in the other party’s interest to prevent that threat, so the partner can continue to provide whatever benefit it was providing (e.g. sexual access, parental effort, co-foraging, predator detection and metabolic byproducts).

One important source of stake is an existing reciprocal relationship [43,45]. When two organisms engage in reciprocal cooperation, they benefit from their partner’s continuing presence so that the reciprocity can continue. In two mathematical models, Barclay [43] showed that it pays to help a reciprocal partner—even anonymously if necessary—if doing so makes that partner more likely to remain available (e.g. alive and uninjured). Pleasant and Barclay [45] tested this in a series of experiments using a Prisoner’s Dilemma with an anonymous opportunity for one partner to help the other after rounds: they showed that people are more likely to help a cooperative partner if doing so keeps that partner in the experiment than if it just increases the partner’s payoff, but only so for cooperative partners (i.e. not for defectors or people with whom they did not interact). The longer the expected interaction, the more costs an organism should be willing to incur [43]. This principle may explain a key feature of human friendships: what starts out as surface-level reciprocity can develop into deep concern for a friend, such that people no longer actively track reciprocity [46].

However, one only has a stake in a partner who cannot be easily replaced [39]. If one can easily choose a new partner to replace an incapacitated one, then one should be willing to incur less cost to save the existing partner [43]. In experiments [45], people were less willing to save an existing partner if there was a good alternative available than if there were no alternatives or only a bad alternative. Thus, organisms have less stake in their partners if they can easily choose another good partner.

### 1.2. Integrating the two perspectives

According to the above, there are two opposing perspectives in the literature: one saying that cooperation is higher when organisms can choose partners (e.g. [5,8,15,19,23,27,28]), and another saying that cooperation is lower when organisms can choose partners [39,43,45]. How can we reconcile these perspectives?

The key to reconciling these perspectives is that some forms of cooperation rely on observation but others do not (e.g. [42,47]). For example, direct reciprocity, indirect reciprocity and costly signalling all rely on observation: someone—either the recipient or a third party—observes an agent’s cooperation and responds in a way that benefits that agent (e.g. reciprocation, alliances, trust, deference, mating; see [10]). Unobserved cooperation brings no reciprocal or signalling benefit and is typically seen as a byproduct of a cooperative sentiment that is ‘adaptive on average’ [42,48]. By contrast, other forms of cooperation require no observation: kinship, stake, mutualism and helping in a Volunteer’s Dilemma. For example, it pays to help one’s genetic kin regardless of whether anyone sees, and it pays to protect the shade-producing tree even if it has no means of detecting your help. Reputation can make one *more* willing to help in these circumstances [47], but is not strictly necessary.

I propose that partner choice promotes observed cooperation but inhibits unobserved cooperation. Partner choice makes it more important to cooperate when observed: cooperation keeps one’s reciprocal partners from leaving, and it signals valuable qualities about oneself to attract and be trusted by new partners. By contrast, partner choice undermines one’s stake in one’s partners, because one can always replace them, so one has less incentive to help if unobserved. A similar principle has been hinted at with genetic relatedness: parents are less concerned for any given offspring if they have other offspring to invest in (e.g. [49]), including the choice to have future offspring. In this article, I provide a model showing that when organisms can choose a new partner, it makes them less likely to provide unobserved stake-based help but more likely to provide observed reciprocity-based help.

## 2. Model and results

### 2.1. Basic setup of model

Imagine two organisms cooperating reciprocally in multi-round Prisoner’s Dilemma, where it costs *c* to confer benefit *b* upon one’s partner. After any given round, there is a probability *w* of another round with the same partner, regardless of how many rounds the pair has been together. As such, at the start of any round, the pair can be expected to last an average of 1/(1 *− w*) more rounds, including the current round. For a conditional cooperator (i.e. someone who cooperates on the condition that their partner does likewise), having a cooperative partner is worth *b – c* in each of the 1/(1 *− w*) remaining rounds, whereas an uncooperative partner provides no benefits and is thus worth zero. An organism without a partner pays no cost and receives no benefit.

Suppose that in a random round, one’s partner is about to die or otherwise become unavailable to cooperate, due to injury, incapacitation, emigration, bankruptcy and so on; for simplicity, I will collectively refer to any of these as a partner becoming ‘unavailable’ or ‘failing to survive’. Now suppose that one could ‘save’ that partner by helping them at a one-time cost *a*, which then keeps them available (e.g. alive, uninjured, capable and solvent). In such a case, it is worth paying the cost of helping whenever (*b − c*)/(1 *− w*) > *a*. This helping is not out of reciprocity, nor is it ‘reciprocated’ by the partner, and in fact, need not be observed—the benefit is that one’s partner stays alive and available to continue the existing reciprocal relationship. This is a very general model with few assumptions; see Barclay [43] for details and an examination of those assumptions.

### 2.2. Adding partner choice

Now imagine that there is a third party present. If one’s partner becomes unavailable (e.g. dead and incapacitated) with probability 1*− w*, then one can choose to interact with that third party instead with probability *f*. For simplicity, the main model below assumes that this third party is also a conditional cooperator like the first partner; the electronic supplementary material shows that the main conclusions are the same when we relax these assumptions (electronic supplementary material, S1).

Furthermore, to make the ‘saving’ more general, I let the help vary in its effectiveness. Instead of being guaranteed to save a partner, let the help be such that one can pay *a* to temporarily increase one’s partner’s availability by Δ*w*, either by preventing a partner’s loss (lower *w*) or by providing them with an enhancement (higher *w*); for simplicity, I use the latter in the payoff equations below, but the end result (Inequality 1) is identical if I use the former.

#### 2.2.1. Unobserved helping

First, let us examine the case where one’s helping is unobserved, such that neither one’s partner nor the third party will become aware of it. In this case, the main benefit of helping is that it keeps a partner alive and available for an expected 1/(1 *− w*) future rounds, though if they become unavailable, then with probability *f* one can interact with the third party instead for those 1/(1 *− w*) future rounds. The payoff for not helping when unobserved (*π_nu_*) is:


(5.1) $$\pi_{nu}=\;w\left(\frac{b-c}{1-w}\right)+\;\left(1-w\right)f\left(\frac{b-c}{1-w}\right).$$

In other words, if the partner survives the focal round on its own with probability *w*, then it continues to be available and provides benefits (*b − c*) for 1/(1 *− w*) more rounds. If the partner does not survive on its own (1 *− w*), then one can interact with the third party instead, with probability *f,* and get the benefits of cooperating (*b − c*) with them for 1/(1 *− w*) more rounds.

By contrast, the payoff for helping when unobserved (*π_hu_*) is:


(5.2) $$\pi_{hu}=\;\left(w+\Delta w\right)\left(\frac{b-c}{1-w}\right)+\;\left(1-w-\Delta w\right)f\left(\frac{b-c}{1-w}\right)-a.$$

In this case, the partner has an increased probability of surviving the focal round (*w* + Δ*w*) to be available to provide benefits (*b − c*) for 1/(1 *− w*) rounds, but at cost *a*; if the partner does not survive (1 *− w* − Δ*w*), then one can interact with the third party instead with probability *f* and cooperate with them. When unobserved, the payoff for helping outweighs the payoff for not helping (*π_hu_* > *π_nu_*) when:


$$\left(1-f\right)\frac{\Delta w}{1-w}\left(b-c\right)>a\;\text{(Inequality 1)}$$

**Figure 1.**
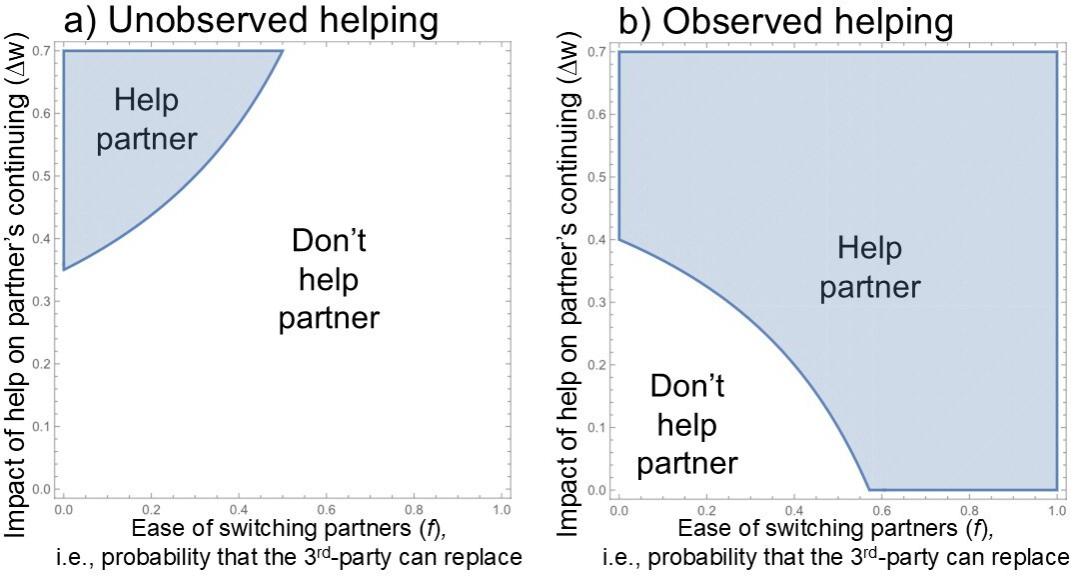
Effect of partner choice (*f*) on (a) unobserved helping and (b) observed helping. Shaded areas represent the parameter regions where it pays to help one’s partner—unobserved helping is more likely to pay off as *f* decreases, whereas observed helping is more likely to pay off as *f* increases. Note: observed helping is always more likely to pay off than unobserved helping (see text), so to show the different effects of *f* within a comparable range of *f*-values, the two panels graph different values of *b*; panel a has *b* = 3 and panel b has *b* = 2. Other parameters displayed are *a* = 1, *c* = 1, *w* = 0.3.

According to Inequality 1, helping pays off less when *f* is high, i.e. when one can easily choose to interact with a third party instead of one’s currently unavailable partner (figure 1a). In other words, when partner choice is more feasible, one has less stake in the well-being of one’s current partner. By contrast, helping pays off more when it has a larger impact on the partner’s availability (high Δ*w*), when the interaction has a high baseline probability of continuing (high *w*), when there are large gains from cooperation (high *b* and/or low *c*), or when the costs of helping are low (low *a*).

#### 2.2.2. Observed helping

Next, we examine the case where one’s helping is observed, such that both one’s partner and the third party will become aware of it and respond accordingly. In this case, one’s help not only keeps one’s partner available, but it also helps keep them (and the third party) cooperating. In many cases, observers will treat a failure to help as a defection and will respond with defection in the Prisoner’s Dilemma. However, rather than just assume this, I allow this to vary: if one is observed to not help, then with probability *d* the partner and the observer respond with defection, and with probability 1 *− d* they ignore the not-helping and continue to cooperate. In other words, they only link one’s helping behaviour with the Prisoner’s Dilemma with probability *d*, which can range from 0 (not-helping is never judged poorly) to 1 (not-helping is treated exactly like defection in the Prisoner’s Dilemma), with intermediate ranges possible (e.g. some observers are more likely to judge negatively, cognitive limitations make the linking not guaranteed, not-helping is judged intermediately resulting in a partial loss of cooperation).

In this situation, the payoff for not helping when observed (*π_no_*) is:


(5.3) $$\pi_{no}=\left(1-d\right)\left(w\left(\frac{b-c}{1-w}\right)+\;\left(1-w\right)f\left(\frac{b-c}{1-w}\right)\right).$$

In other words, if the focal agent is observed to not help, then it only earns anything if its partner and the third party do not link helping with the Prisoner’s Dilemma (1 *− d*). For simplicity, this assumes that if the partner (or observer) ceases cooperating, then they do so forever. The electronic supplementary material relaxes this assumption and allows a focal agent to restart reciprocity by unilaterally cooperating and allowing itself to get suckered in one round (electronic supplementary material, S2); the results below are qualitatively similar.

If one helps, then one’s partner and the observer cooperate in return, so the payoff for helping when observed (*π_ho_*) is:


(5.4) $$\pi_{ho}=\;\left(w+\Delta w\right)\left(\frac{b-c}{1-w}\right)+\;\left(1-w-\Delta w\right)f\left(\frac{b-c}{1-w}\right)-a.$$

As such, when observed, the payoff for helping outweighs the payoff for not helping (*π_ho_ > π_no_*) when:


$$\left(\Delta w\left(1-f\right)+d\left(w+\left(1-w\right)f\right)\right)\frac{b-c}{1-w}>a\;\text{(Inequality 2)}$$

Which can be rearranged in terms of partner choice (*f*) as:


$$\left(f\left(d\left(1-w\right)-\Delta w\right)+dw+\Delta w\right)\frac{b-c}{1-w}>a\;\text{(Inequality 3)}$$

According to Inequality 3, observed helping generally pays off more when partner choice *f* is high (i.e. when one can easily choose to interact with a third party instead of one’s currently unavailable partner, see figure 1b). This positive effect of partner choice occurs as long as others are likely to treat a failure to help as a defection and respond accordingly (i.e. high enough *d*). For example, when *d* = 0 (i.e. no linkage between helping and the Prisoner’s Dilemma), Inequality 3 simplifies to Inequality 1—observed helping has as little effect on anyone’s behaviour as unobserved helping, such that the ability to choose a new partner inhibits helping. By contrast, when *d* = 1 (i.e., full linkage), Inequality 3 simplifies to:


$$\left(f\left(1-w-\Delta w\right)+w+\Delta w\right)\frac{b-c}{1-w}>a\;\text{(Inequality 4)}$$

In other words, when there is full linkage between helping and the Prisoner’s Dilemma (i.e. *d* = 1), it pays to help so that one receives cooperation ((*b − c*)/(1 *− w*)) from either a current partner who survives (*w* + Δ*w*) or from an observing third party that one can choose instead (*f*(1 *− w* + Δ*w*)).

How strong does the linkage need to be for partner choice to promote observed helping? By examining Inequality 3, we see that the ability to choose a new partner (*f*) facilitates observed cooperation whenever *d*(1 *− w*) − Δ*w* > 0, which can be rearranged as:


$$d>\frac{\Delta w}{1-w}\;\text{(Inequality 5)}$$

In other words, for partner choice to promote cooperation, the linkage (*d*) needs to be stronger than the proportional effect of helping, i.e. how much of the risk of incapacitation (1 *− w*) that the helping (Δ*w*) will alleviate.

Inequalities 2 and 3 also show some more predictable results: observed helping pays off more when others judge you for not helping (high *d*), when the helping has more impact on partner (high Δ*w*), when interactions are more likely to continue (high *w*), and when there are more gains from cooperation (high *b* or low *c*).

Figure 1 illustrates how partner choice (*f*) makes unobserved helping less likely (panel a) and observed helping more likely (panel b). The rationale is simple: On the one hand, partner choice gives you an incentive to help publicly, so that observers will see it, just in case you need to switch to those observers for any reason (e.g. incapacitation of current partner). On the other hand, partner choice means you have less stake in your existing partner, and therefore less reason to help them—especially when unobserved—because you can easily replace them with a new partner (see also [43,45]).

## 3. Discussion

This model shows that partner choice has mixed effects on cooperation: it decreases anonymous (stake-based) cooperation, but usually increases observable (reputation-based) cooperation. This finding is robust to several changes in assumptions, such as whether new partners might be defectors (electronic supplementary material, S1), if one can restore one’s reputation after being seen to not help (electronic supplementary material, S2), whether observability is a continuous instead of binary trait (electronic supplementary material, S3), and whether one can have more than one partner (electronic supplementary material, S4).

These results highlight a negative impact of partner choice: when organisms can choose new partners, they have less stake in their current partners and have less reason to protect them. This may lead to a superficial form of cooperation: cooperate solely to be seen, but when one’s partner really needs help, switch partners rather than stay and pay the cost to help. In other words, partner choice exacerbates the ‘Banker’s Paradox’ of those who most need help being ‘the worst credit risks’ [39]. By contrast, one has the most stake in a current partner if one cannot easily choose to replace them, either because partner choice is impossible or costly, or because one’s current partner provides benefits that are irreplaceable.

### 3.1. Effects of partner choice on observable cooperation

The model also produces a counterintuitive result about observed cooperation: although partner choice will usually make organisms more likely to cooperate when observed, there are some circumstances where it will not. The net effect of partner choice depends on the balance of two factors: the amount it reduces one’s stake in a current partner versus the benefits of having others see you cooperate. What affects the relative importance of each of these?

Reputational benefits are more important when the observers would comprise a larger fraction of one’s future interactions. For example, if one’s current partner is less likely to survive (low *w* or Δ*w*), then one is more likely to interact with a third-party observer than with one’s current partner. As such, with low-continuation interactions, the presence of a third party makes it more worthwhile to be seen to cooperate. Also, if an agent can interact with both the current partner and the observer (i.e. partners are additive instead of replacements), then this provides an additional reason to be seen to cooperate, so the presence of a third party increases cooperation even more (electronic supplementary material, S4). In such cases, partner choice will increase observed cooperation.

By contrast, reputation is less important when there are only small gains from being seen to cooperate. For example, if no one judges non-helpers harshly (low *d*), or when one can easily restore one’s reputation for not helping (electronic supplementary material, S2), then partner choice has little beneficial effect on cooperation. In such cases, partner choice reduces observed cooperation (in addition to reducing anonymous cooperation), because the presence of replacement partners makes one less willing to pay to save one’s existing partner. Future work should examine when others will treat non-helping as defection versus ignore it.

All this being said, we must not confuse the effects of observation with the effects of partner choice—these are different factors. In the model, observability always increased cooperation—there were no cases where observed helping was lower than unobserved helping. Instead, it is only partner choice that has mixed effects on cooperation.

### 3.2. Distinguishing stake-based cooperation from reputation-based cooperation

These results also highlight the need for researchers to differentiate between observed and anonymous cooperation, because these may have very different causes. Traditionally, many researchers have assumed that anonymous cooperation is produced by a psychology that evolved for reputation-based cooperation, but which acts under uncertainty—it pays to cooperate just in case others discover one’s actions (e.g. [42,48,50,51]). However, the present results show that anonymous cooperation can also be caused by a psychology that evolved for assessing and acting on one’s stake in others, and that such a psychology can respond differently than a psychology for reputation-based cooperation. More generally, the present results show that it’s useful to distinguish between cooperation that relies on observation (e.g. reciprocity, costly signalling) and cooperation that does not (e.g. stake and kinship, Volunteer’s Dilemma; see [47]. If we seek to promote cooperation based on factors that increase reputation-based cooperation, it may backfire for other kinds of cooperation, and vice versa.

Partner choice varies across cultures and individuals; for example, some cultures provide more opportunity for people to change social partners (high ‘relational mobility’; e.g. [30,52]), and some individuals are more desirable in the ‘biological market’ for partners (e.g. [23,24]). Relational mobility is associated with higher general trust (e.g. [30]), but the present model reveals a potential downside—people with higher relational mobility have less stake in their partners. The current model predicts that countries with high relational mobility may show more overt reciprocity- or signaling-based cooperation but will have lower levels of discreet stake-based cooperation or of truly valuing one’s partners. As tentative support, Horita *et al*. [29] recently showed that relational mobility predicts generalized trust but not intimacy with friends.

Organisms should try to distinguish between stake-based cooperation and observation-based cooperation: a partner who genuinely values our welfare (i.e. high stake) is obviously more valuable than a partner who only cooperates so we see it (i.e. observation-based). Organisms may distinguish between these two types of cooperators using a number of cues. One such cue is if a partner’s low-observability helping just happens to be discovered. This indicates that the partner directly values one’s welfare, or they value the future cooperation enough to reciprocate even if discovery is low [53].

Other cues of stake could include traits or behaviour that reduce one’s ability to switch partners. For example, high courtship costs, initiation rituals, legal contracts, disclosure of secrets, cohabitation or joint endeavours make it costly to switch partners and thus increase one’s stake in a current partner. Similarly, if friendships or romances only develop slowly (e.g. ‘Raise the Stakes’ [54,55]), then switching partners involves an opportunity cost, such that one has more stake in a current partner. More generally, because they indicate a reduction in one’s outside options, observers should treat signals of commitment, whether emotional or behavioural (e.g. [23,56–58]), as signals of genuinely valuing a partner. And as previous research shows, individuals should trust and prefer others who appear to have a stake in them (e.g. [59]).

Third parties might even use the wellbeing of an agent’s partner as a cue of that agent’s cooperation. If a focal agent’s partner remains in better condition than one should expect based on the agent’s observed cooperation, then that might indicate that the focal agent helps the partner when unobserved. For example, if A’s partner(s) seem to all be in better-than-expected condition, but B’s partner(s) seem to die or get injured, then one might infer that A is a better partner than B, either because A saves its partners unobserved or because B harms its partners. Such an idea is beyond the scope of the current paper, but would be interesting to examine in the future—whether organisms assess each others’ value as a partner by evaluating the wellbeing of each others’ partners (though see also assortative pairing based on market value [23,24].

### 3.3. Limitations and conclusions

This is a very simple model with few assumptions, so it does not apply to any one particular scenario. Rather than be a limitation, that is a strength, because it increases the model’s generality. For example, the model applies whether the cost and benefit parameters represent lifetime fitness, survival, fecundity or some proxy thereof. It also does not matter whether one’s partner becomes unavailable due to death, injury, illness, incapacitation, emigration, bankruptcy or any other preventable reason. One specific assumption is that the model assumes a partner becomes completely unavailable. However, the general principle applies even if a partner is only partly incapacitated—a fraction of the incapacitation warrants a fraction of the cost to preserve that partner’s full availability, and that fraction of a stake is reduced if one can switch to a fully capable partner.

A second assumption is that the model uses specific types of reciprocators, such as Tit-for-Tat. With more complex cooperative strategies, the exact equilibria can vary slightly, but this does not change the underlying point that partner choice affects stake-based cooperation differently from reciprocity-based cooperation (e.g. see electronic supplementary material, S2). Similarly, the presence of errors will affect the exact equilibria, but not the main point about partner choice’s effects on those equilibria.

A third assumption in the model is that there is only one replacement partner. In reality, the more observers there are, the more incentive there is to appear cooperative (e.g. [3]); this increases the reputational benefits and makes partner choice more likely to increase overall cooperation. As the electronic supplementary material, S4 shows, if one can interact with multiple partners, then partner choice has a more positive net effect.

A fourth assumption of the model is that all cooperators are equally valuable. In reality, some partners are more willing or able to provide benefits (e.g. [23,24,60]). If one’s current partner is much better than others, then that partner cannot easily be replaced, so the presence of others will have little effect (unless one can attract additional partners). By contrast, if one’s current partner is substandard, then one might even benefit from their demise if that makes it easier to switch partners (e.g. [43]). Correspondingly, Pleasant and Barclay [45] found that people were less likely to anonymously help an uncooperative partner if there was another cooperator present. However, the presence of a better partner should still promote observed cooperation if doing so affects one’s likelihood of being chosen or of receiving cooperation from the high-quality observer.

Like previous models on stake [43], organisms have a stake in their partners’ ability to cooperate (e.g. availability, wellbeing, capabilities), but not their partners’ reproduction. More generally, a partner’s payoff in some fitness proxy is only valuable insofar as it contributes to that partner’s future ability to cooperate. In support of this, Pleasant and Barclay [45] showed that participants in an economics experiment will pay to anonymously help a cooperative partner if doing so allows that partner to keep playing the cooperative game, but they do not particularly pay to anonymously increase that partner’s earnings—the partner’s presence is valuable, but the partner’s payoff is not. As such, it is slightly misleading to refer to one’s stake in a partner as ‘fitness interdependence’ (cf. [40]), because we have a stake in their availability and wellbeing but not their fitness *per se* (i.e. reproduction). Future work can examine the cases where partnerships persist across multiple generations, which is where one would have a stake in a partner’s reproduction.

Partner choice is powerful at promoting cooperation, but the present results show that it also undermines cooperation by reducing individuals’ stake in their partners. As such, partner choice promotes observable reputation-based cooperation but reduces anonymous stake-based cooperation. The net effect of partner choice depends on the relative importance of these two factors. We look forward to future work that distinguishes between these different forms of cooperation.

## Data Availability

This article has no additional data [61].
